# Dietary protocatechuic acid ameliorates inflammation and up-regulates intestinal tight junction proteins by modulating gut microbiota in LPS-challenged piglets

**DOI:** 10.1186/s40104-020-00492-9

**Published:** 2020-09-09

**Authors:** Ruizhi Hu, Ziyu He, Ming Liu, Jijun Tan, Hongfu Zhang, De-Xing Hou, Jianhua He, Shusong Wu

**Affiliations:** 1 Hunan Collaborative Innovation Center for Utilization of Botanical Functional Ingredients, College of Animal Science and Technology, Hunan Agricultural University, Changsha, 410128 China; 2 Department of Food Science and Biotechnology, Faculty of Agriculture, Kagoshima University, Kagoshima, 890-0065 Japan; 3 Beijing China-Agri HongKe Bio-Technology Co., Ltd., Beijing, 102206 China; 4 State Key Laboratory of Animal Nutrition, Institute of Animal Sciences, Chinese Academy of Agricultural Sciences , Beijing, 100193 China

**Keywords:** Gut microbiota, Inflammation, Piglets, Protocatechuic acid, Tight junction proteins

## Abstract

**Background:**

Weaning is one of the major factors that cause stress and intestinal disease in piglets. Protocatechuic acid (PCA) is an active plant phenolic acid which exists in Chinese herb, Duzhong (*Eucommia ulmoides* Oliver), and is also considered as the main bioactive metabolite of polyphenol against oxidative stress and inflammation. This study aimed to investigate the effect of PCA on growth performance, intestinal barrier function, and gut microbiota in a weaned piglet model challenged with lipopolysaccharide (LPS).

**Methods:**

Thirty-six piglets (Pig Improvement Company line 337 × C48, 28 d of age, 8.87 kg ± 0.11 kg BW) were randomly allocated into 3 treatments and fed with a basal diet (CTL), a diet added 50 mg/kg of aureomycin (AUR), or a diet supplemented with 4000 mg/kg of PCA, respectively. The piglets were challenged with LPS (10 μg/kg BW) on d 14 and d 21 by intraperitoneal injection during the 21-d experiment. Animals (*n* = 6 from each group) were sacrificed after being anesthetized by sodium pentobarbital at 2 h after the last injection of LPS. The serum was collected for antioxidant indices and inflammatory cytokines analysis, the ileum was harvested for detecting mRNA and protein levels of tight junction proteins by PCR and immunohistochemical staining, and the cecum chyme was collected for intestinal flora analysis using 16S rRNA gene sequencing.

**Results:**

Dietary supplementation of PCA or AUR significantly increased the expression of tight junction proteins including ZO-1 and claudin-1 in intestinal mucosa, and decreased the serum levels of thiobarbituric acid reactive substances (TBARS) and IL-6, as compared with CTL group. In addition, PCA also decreased the serum levels of IL-2 and TNF-α (*P* < 0.05). Analysis of gut microbiota indicated that PCA increased the Firmicutes/Bacteroidetes ratio (*P* < 0.05). Spearman’s correlation analysis at the genus level revealed that PCA reduced the relative abundance of *Prevotella* 9, *Prevotella* 2, *Holdemanella*, and *Ruminococcus torques* group (*P* < 0.05), and increased the relative abundance of *Roseburia* and *Desulfovibrio* (*P* < 0.05), whereas AUR had no significant effect on these bacteria.

**Conclusions:**

These results demonstrated that both PCA and AUR had protective effect on oxidative stress, inflammation and intestinal barrier function in piglets challenged with LPS, and PCA potentially exerted the protective function by modulating intestinal flora in a way different from AUR.

***Holdemanella*:**

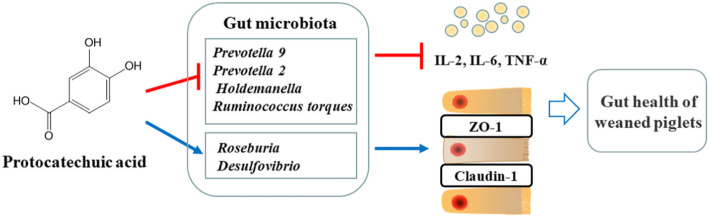

## Background

The intestine is not only the main site of digestion and nutrients absorption but also the first immune barrier against food-derived pathogens. Thus, maintaining intestinal integrity and functions is essential for human and animal health. In swine production, the gastrointestinal tract of neonatal piglets is vulnerable to diarrhea post-weaning [1, 2], which can directly cause the intestinal system dysfunctions and results in intestinal barrier injury, intestinal inflammation, oxidative damage and microbial disorder [1, 3]. Previous studies indicated that the intestinal microbiome plays a very important role in maintaining intestinal function and host health, and an appropriate intestinal bacterial community can resist the infection of pathogens and improve mucosal immunity [4]. Thus, gut microbiota modulation has become a new strategy to improve intestinal health.

For decades, antibiotics are used as growth promoters and to protect against pathogens in the swine industry. However, due to the concern of the increasing bacterial resistance, nutritional strategies are expected to be an alternative of antibiotics [4]. Polyphenols possess multiple biological functions such as antimicrobial [5], anti-inflammatory [6], antioxidant [7] and antiviral [8]. However, polyphenols are hard to be directly absorbed in the stomach or small intestine, and most of them are moved into the hindgut and further metabolized by microbiome [9, 10], which suggests that the interaction between polyphenols (including their metabolites) and gut microbiota is critical to understanding the biological mechanisms of polyphenols. Protocatechuic acid (PCA, 3,4-dihydroxybenzoic acid) is an active phenolic acid existing in many plants such as Chinese herb Duzhong (*Eucommia ulmoides* Oliver) [11], alpinia [12], and ilex [13]. Moreover, PCA is also considered as the major bioactive metabolite of polyphenols, especially anthocyanins [14]. Previous studies have reported that PCA possesses antioxidant [15], anti-inflammatory [16], antibacterial [17] and antiviral [18] effects and has the potential to attenuate intestinal injury [9, 19]. Meanwhile, intestinal flora disturbance is reported to be associated with intestinal injury, inflammation, and impaired barrier function [20], and our previous study has indicated that polyphenols may exert beneficial biological functions by regulating the gut microbial community [5]. Thus, in the present study, we challenged to clarify the effect of PCA on gut microbiota and their correlation with intestinal barrier function in lipopolysaccharide (LPS)-challenged weaned piglets.

## Methods

### Materials and reagents

Protocatechuic acid (≥ 98%) was provided by Shanghai Yuanye Bio-Technology Co., Ltd. (Shanghai, China). LPS (*Escherichia coli* Serotype O55:B5) was purchased from Sigma-Aldrich (St. Louis, MO, USA). Antibodies against occludin, claudin-1, ZO-1, and corresponding secondary antibodies were purchased from Proteintech Group, Inc. (Chicago, IL, USA). Aureomycin (AUR) and ingredients for basal diet were provided by Hunan Liuyanghe Feedstuff Co., Ltd. (Changsha, Hunan, China).

### Experimental design and diets

The animal model and experimental procedures used in this experiment were approved by the Hunan Agricultural University Institutional Animal Care and Use Committee. A total of 36 piglets (Pig Improvement Company line 337 × C48, 28 d of age, 8.87 kg ± 0.11 kg BW), which were housed in the fully slatted pens and had free access to feed and water, were randomly allocated into 3 treatments with 6 replicate pens per treatments and 2 barrows per pen. Piglets in the treatments were fed with a basal diet (control group, CTL), a diet added 50 mg/kg of aureomycin (AUR group), or a diet supplemented with 4000 mg/kg of PCA (PCA group), respectively, for 21 d. All piglets were challenged with LPS (10 μg/kg BW) on d 14, and d 21 (2 h before sacrifice) through intraperitoneal injection. The dosage of PCA was based on previous studies [21, 22]. The composition of the basal diet, which meets the NRC (2012) Nutrient Requirements of Swine, was shown in Supplemental Table 1.

### Growth performance

The BW of piglets was individually measured at the morning of d 1 and d 21 after fasting overnight, and feed intake per pen was collected daily throughout the trial to calculate average daily feed intake (ADFI), average daily gain (ADG), and feed/gain ratio (F/G).

### Sample collections

Six piglets (one with average body weight from each replicate pen) from each group were sacrificed after being anesthetized with sodium pentobarbital. Blood samples (10 mL) were collected from the jugular vein into anticoagulant-free vacuum tubes and centrifuged at 1500×*g* for 10 min after standing at room temperature for 30 min to get the serum. Cecum contents were collected immediately into sterile tubes and snap-frozen in liquid nitrogen before storage at − 80 °C for further DNA extraction. Two segments (around 2 cm long) of ileum were gently washed in normal saline, one was immediately fixed in phosphate-buffered paraformaldehyde (4%, pH 7.6) for immunohistochemical staining of tight junction proteins, and the other was used for collecting mucosal scrapings for detecting their mRNA expressions.

### Measurement of antioxidant indices and inflammatory cytokines in serum

Total antioxidant capacity (T-AOC), Total superoxide dismutase (T-SOD) activity, glutathione peroxidase (GSH-Px) activity, and the level of thiobarbituric acid reactive substances (TBARS), an indicator of oxidative stress, were determined in serum by using respective assay kits (Nanjing Jiancheng Bioengineering Institute, Nanjing, China) according to the manufacturer’s instructions as described previously [23]. Serum levels of interleukin (IL)-1β, IL-2, IL-6, and tumor necrosis factor-α (TNF-α) were measured with respective ELISA kit (Elabscience Biotechnology Co., Ltd., Wuhan, Hubei, China) according to the manufacturer’s manual.

### Real-time PCR

The mRNA expression of tight junction proteins including occludin, claudin-1, and ZO-1 in ileum mucosa was determined by real-time quantitative PCR. Briefly, total RNA was isolated using the TRIzol Reagent (Sangon Biotech, Shanghai, China) according to the manufacturer’s instruction, and the purity of total RNA was identified spectrophotometrically via usage of optical density (OD) 260 nm and 280 nm measurements (Merinton Instrument, Inc., Ann Arbor, MI, USA). The real-time PCR was performed as described previously [24], β-actin was used as a housekeeping gene to normalize target gene transcript levels and the primers of genes (Sangon Biotech, Shanghai, China) are shown in Supplemental Table 2. The PCR reactions were performed in a 20-μL total reaction volume, which included 10 μL of 2 × SybrGreen qPCR Master Mix (Thermo Scientific), 0.4 μL each of the forward and reverse primers (10 μmol/L), 2 μL of cDNA template, and 7.2 μL of sterilized water. The PCR was carried out on a LightCycler480 Real-Time PCR system (Rotkreuz, Switzerland). The thermal cycler parameters were as follows: 3 min at 95 °C, 45 cycles for 5 s at 95 °C, 30 s at 6 °C. The stability of the β-actin genes was evaluated by measuring the fluctuation range of the Ct values.

### Immunohistochemical staining

The segment of ileum was paraffin-embedded and cut into 5-μm sections before transfer to glass slides. The paraffin sections were then placed in an oven at 37 °C for 12 h. After deparaffinization in xylene for 20 min × 3 times, the sections were then soaked in 100%, 95%, 85%, 75% of ethanol and distilled water for 5 min successively. The sections were boiled in 0.01 mol/L citrate buffer (pH 6.0) for 22 min, and then cooled to room temperature. After cooling, the sections were washed with 0.01 mol/L PBS (pH 7.2–7.6) for 3 min × 3 times, and then soaked in 3% H_2_O_2_ for 15 min before being washed with PBS for 3 min × 3 times. Sections were next incubated with specific primary antibody (occludin, claudin-1 or ZO-1 with a dilution rate of 1:100) at 4 °C overnight, followed by incubation with corresponding HRP-conjugated secondary antibody at 37 °C for 30 min. The sections were washed with PBS for 5 min × 3 times after each incubation, and immunostained with DAB chromagen before counterstained with Hematoxylin (Sigma-Aldrich, St. Louis, MO, USA). Finally, the sections were dehydrated in graded alcohol (60–100%, 5 min each) and cleaned in xylene before being cover-slipped in neutral balsam (Sigma-Aldrich, St. Louis, MO, USA). Stained sections were observed by using a Motic BA210T microscope (Motic China Group Co.,Ltd., Xiamen, Fujian, China).

### Characterization of gut microbiota by 16S rRNA gene sequencing

Total DNA was extracted from cecum contents by using a DNA Isolation Kit (MoBio Laboratories, Carlsbad, CA, USA) following the manufacturer’s manual. Purity and quality of the genomic DNA were checked on 0.8% agarose gels. The V3–V4 hypervariable region of the bacterial 16S rRNA gene was amplified with the primers 338F (5′-ACTCCTACGGGAGGCAGCA-3′) and 806R (5′-GGACTACHVGGGTWTCTAAT-3′). For each cecum content, 10-digit barcode sequence was added to the 5′ end of the forward and reverse primers (provided by Allwegene Technology Inc., Beijing, China). The PCR was carried out on a Mastercycler Gradient (Eppendorf, Germany) using 25 μL reaction volumes, containing 12.5 μL KAPA 2G Robust Hot Start Ready Mix, 1 μL Forward Primer (5 μmol/L), 1 μL Reverse Primer (5 μmol/L), 5 μL DNA (total template quantity is 30 ng), and 5.5 μL H_2_O. Cycling parameters were 95 °C for 5 min, followed by 28 cycles of 95 °C for 45 s, 55 °C for 50 s and 72 °C for 45 s with a final extension at 72 °C for 10 min. Three PCR products per sample were pooled to mitigate reaction-level PCR biases. The PCR products were purified using a QIAquick Gel Extraction Kit (QIAGEN, Germany), and quantified using Real Time PCR, and sequenced on Miseq platform at Allwegene Technology Inc., Beijing, China. After the run, image analysis, base calling and error estimation were performed using Illumina Analysis Pipeline Version 2.6. The raw data were first screened and sequences were removed from consideration if they were shorter than 200 bp, had a low quality score (≤ 20), contained ambiguous bases or did not exactly match to primer sequences and barcode tags. Qualified reads were separated using the sample-specific barcode sequences and trimmed with Illumina Analysis Pipeline Version 2.6. And then the dataset was analyzed using QIIME (Version 1.8.0). The sequences were clustered into operational taxonomic units (OTUs) at a similarity level of 97%, to generate rarefaction curves and to calculate the richness and diversity indices. The Ribosomal Database Project (RDP) Classifier tool was used to classify all sequences into different taxonomic groups.

### Statistical analysis

Results were expressed as means ± SD. The significant differences between groups were analyzed by one-way analysis of variance tests, followed by Fisher’s least significant difference (LSD) and Duncan’s multiple range tests with the SPSS statistical program (SPSS19, IBM Corp., Armonk, NY, USA). A probability of *P* < 0.05 was considered significant.

## Results

### The effect of PCA on growth performance of piglets

The initial BW, final BW, ADG, ADFI and F/G of piglets were shown in Table 1. The ADG of piglets in the CTL group, AUR group and PCA group was 413.75 ± 30.64 g, 436.04 ± 2.67 g and 449.96 ± 43.3 g, respectively. However, there was no significant difference among the three groups.

**Table 1:** The effect of PCA on growth performance of LPS-induced weaned piglets

	CTL	AUR	PCA	*P*-value
Initial BW, kg	8.90 ± 0.15	8.92 ± 0.10	8.86 ± 0.18	0.776
Final BW, kg	17.17 ± 0.53	17.64 ± 0.09	17.86 ± 0.98	0.202
ADG, g	413.75 ± 30.64	436.04 ± 2.67	449.96 ± 43.30	0.153
ADFI, g	598.71 ± 5.48	609.19 ± 2.56	589.19 ± 10.20	0.132
F/G	1.45 ± 0.11	1.39 ± 0.01	1.31 ± 0.12	0.079

CTL, a basal diet; AUR, a basal diet containing 50 mg/kg aureomycin; PCA, a basal diet supplemented with 0.4% protocatechuic acid. Data were shown as means ± SD

### PCA enhanced antioxidant capacity and attenuated inflammation in piglets

**Fig. 1:**
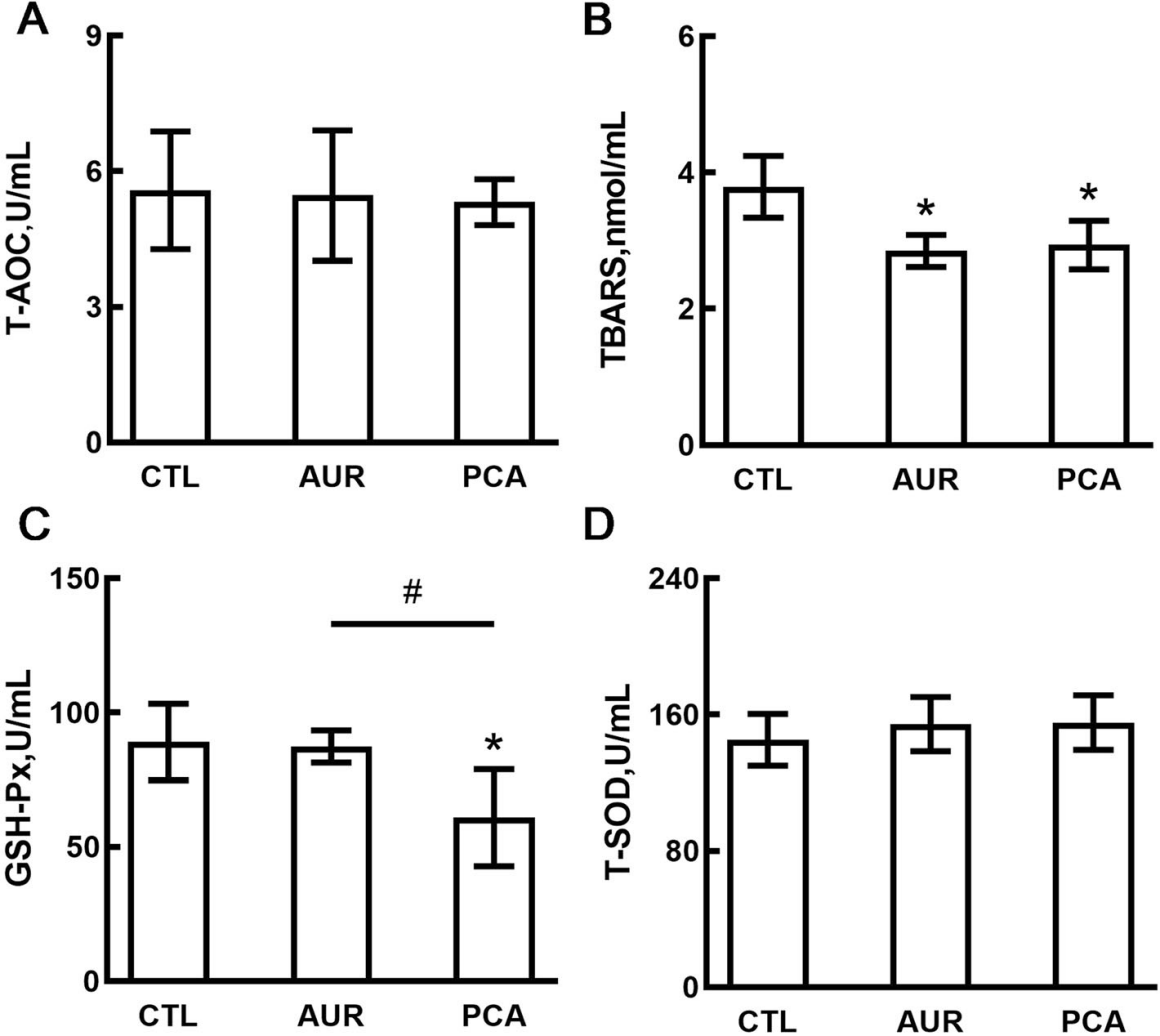
The Effect of PCA on antioxidant indicators. Serum levels of indicators including T-AOC (**a**), TBARS (**b**), GSH-Px (**c**) and T-SOD (**d**) were determined by using respective kits. CTL, a basal diet; AUR, a basal diet containing 50 mg/kg aureomycin; PCA, a basal diet supplemented with 0.4% protocatechuic acid. Data were shown as means ± SD (*n* = 6), *P < 0.05 compared with CTL, ^#^*P* < 0.05 compared with AUR

To understand the effect of PCA on the antioxidant capacity of piglets, antioxidant indicators including T-AOC, TBARS, GSH-Px and T-SOD were measured. As shown in Fig. 1, the level of TBARS (Fig. 1B), an indicator of lipid peroxidation, was significantly decreased in both AUR and PCA groups (*P* < 0.05 compared to CTL group). However, there were no significant differences in T-AOC level (Fig. 1A) and T-SOD activity (Fig. 1D) among the three groups, and the activity of GSH-Px (Fig. 1C) was reduced in the PCA group (*P* < 0.05 compared to CTL or AUR group).

**Fig. 2:**
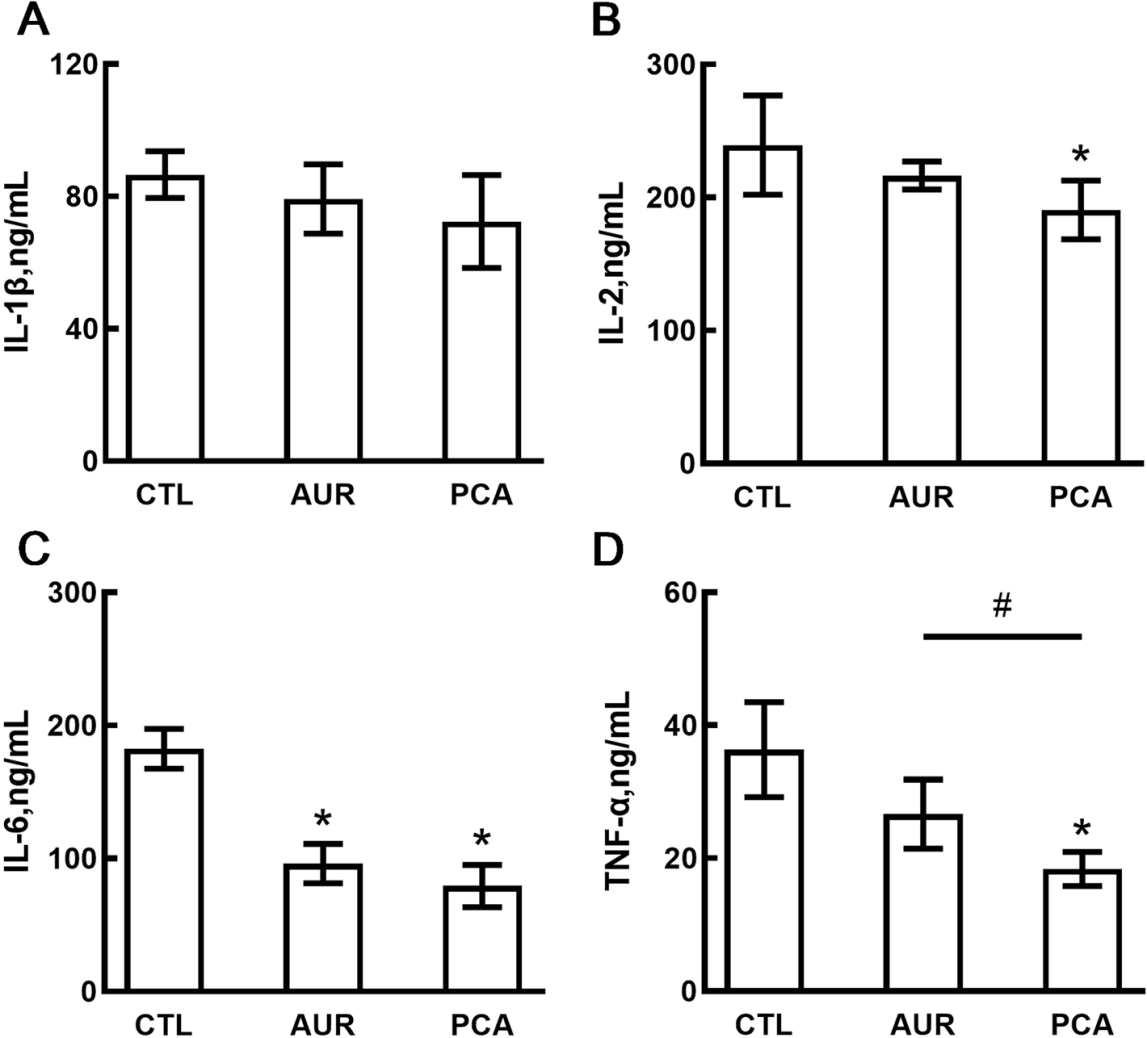
The effect of PCA on the production of inflammatory cytokines. Serum levels of IL-1β (**a**), IL-2 (**b**), IL-6 (**c**) and TNF-α (**d**) were measured by using ELISA kits. CTL, a basal diet; AUR, a basal diet containing 50 mg/kg aureomycin; PCA, a basal diet supplemented with 0.4% protocatechuic acid. Data were shown as means ± SD (*n* = 6), *P < 0.05 compared with CTL, ^#^*P* < 0.05 compared with AUR

Cytokines including IL-1β, IL-2, IL-6 and TNF-α were next measured to reflect the inflammatory status of piglets. Figure 2 showed that serum level of IL-6 (Fig. 2C) was reduced significantly in both AUR and PCA group, while the levels of IL-2 (Fig. 2B) and TNF-α (Fig. 2D) were decreased significantly in PCA group only, as compared with CTL group. There was no significant difference in IL-1β (Fig. 2A) level among the three groups, and no significant difference was observed in IL-1β, IL-2 and TNF-α level between CTL and AUR group. In addition, TNF-α level of PCA group was significantly lower than that of AUR group.

### PCA increased the expression of tight junction proteins

**Fig. 3:**
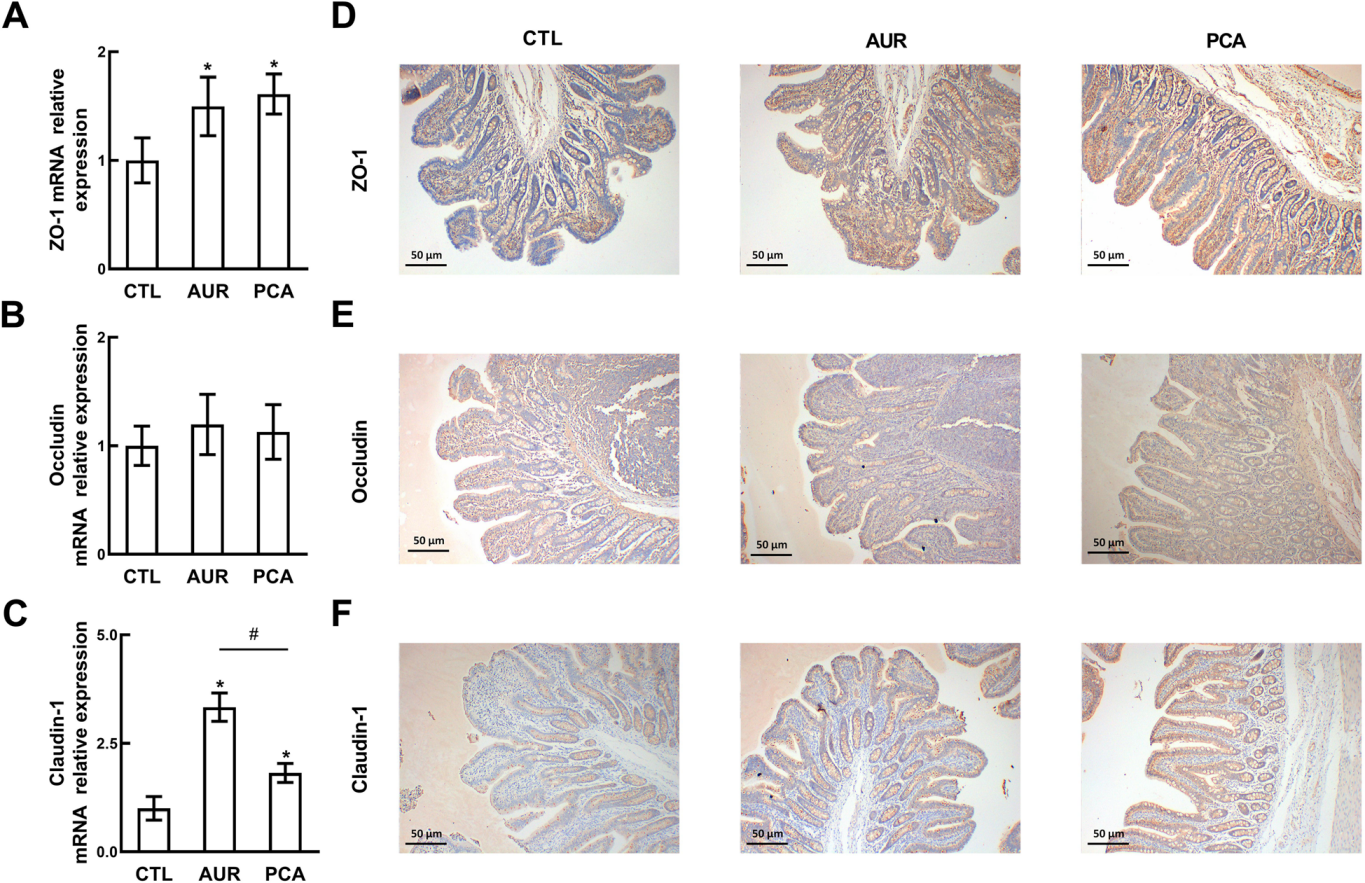
The effect of PCA on the expression of tight junction proteins. The mRNA expression of ZO-1 (**a**), occludin (**b**), and claudin-1 (**c**) was quantitated by real-time PCR, and the protein expression of ZO-1 (**d**), occludin (**e**), and claudin-1 (**f**) was detected by immunohistochemical staining. In real-time PCR, the data were shown as means ± SD (*n* = 6) of mRNA relative expressions. In immunohistochemical staining, the pictures shown were the representative of 6 individual stained sections photographed at 100× magnification, and tight junction proteins were stained yellow or brown-yellow. CTL, a basal diet; AUR, a basal diet containing 50 mg/kg aureomycin; PCA, a basal diet supplemented with 0.4% protocatechuic acid. *P < 0.05 compared with CTL, ^#^*P* < 0.05 compared with AUR

To understand the effect of PCA on intestinal mucosal barrier function, the mRNA expressions of tight junction protein genes including ZO-1, occludin and claudin-1 were measured in ileum mucosa by Real-time PCR, and their proteins were confirmed by immunohistochemical staining. As shown in Fig. 3, dietary supplementation of AUR or PCA significantly increased the mRNA expression of ZO-1 (Fig. 3A) and claudin-1 (Fig. 3C), but not occludin (Fig. 3B), as compared with the CTL group. The immunohistochemical analysis also revealed that AUR and PCA up-regulated the protein levels of ZO-1 (Fig. 3D) and claudin-1 (Fig. 3F), but had limited effect on occludin (Fig. 3E).

### Modulation of gut microbiota by PCA

**Fig. 4:**
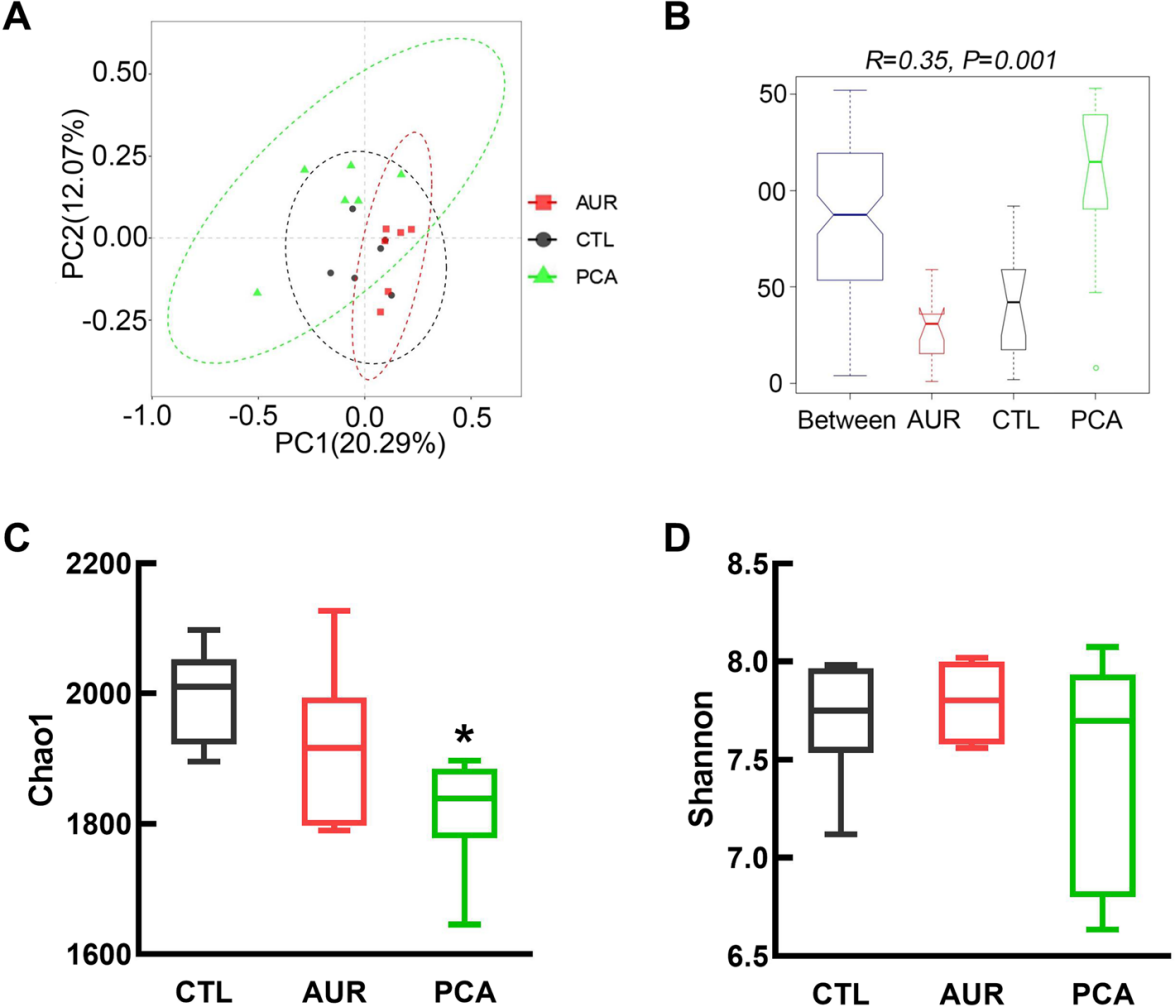
The effect of PCA on cecal microbial community. **a** Principal component Analysis ordination plots of microbial communities in the CTL, AUR and PCA group based on the Bray-Curtis distance metric. **b** Analysis of similarities (ANOSIM) between CTL, AUR and PCA group. **c** The effect of PCA on the chao1 index. **d** The effect of PCA on the Shannon index. CTL, a basal diet; AUR, a basal diet containing 50 mg/kg aureomycin; PCA, a basal diet supplemented with 0.4% protocatechuic acid. Data were shown as means ± SD (*n* = 6), *P < 0.05 compared with CTL

As the microbial community play an important role in gut health and barrier function, the composition of cecal microbiota, which can mirror the microbial colonization in gut, was analyzed by using 16S rRNA gene amplicon sequencing. The principal component analysis (Fig. 4A) and analysis of similarities (ANOSIM) (Fig. 4B) showed that PCA had a significant effect on the cecal microbiota, and there was an apparent difference between the clusters of PCA and CTL group. Further analysis indicated that PCA significantly decreased the species richness (Chao 1, Fig. 4C) as compared with CTL group, while there was no significant difference in the community diversity (Shannon, Fig. 4D) among the three groups.

**Fig. 5:**
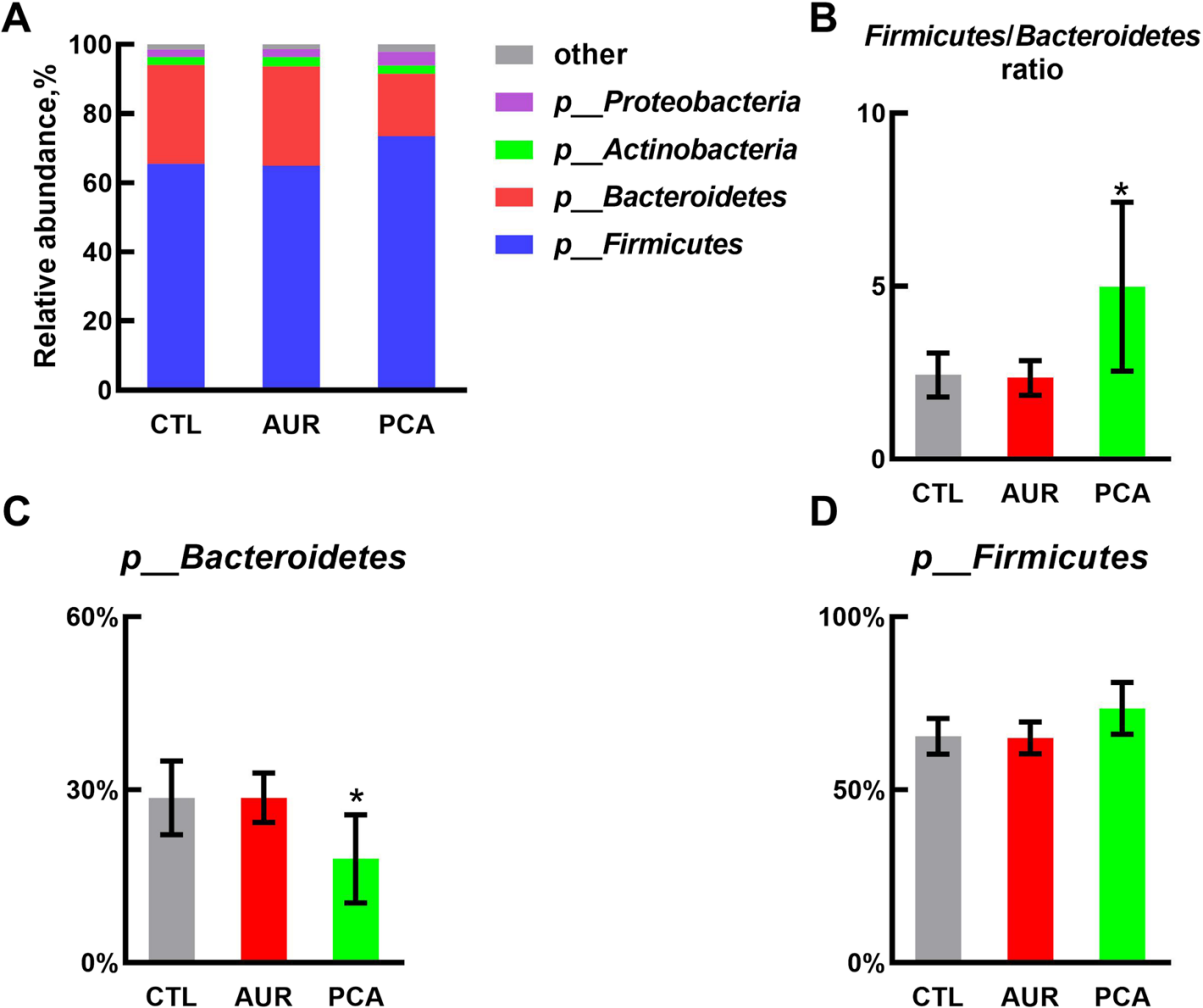
Modulation of gut microbiota by PCA at the phylum level. **a** The relative abundance of cecal microbial phyla (mean of each group). **b** The ratio of Firmicutes to Bacteroidetes in each group based on their relative abundance, and the effect of PCA on Bacteroidetes **c** and Firmicutes **d**. CTL, a basal diet; AUR, a basal diet containing 50 mg/kg aureomycin; PCA, a basal diet supplemented with 0.4% protocatechuic acid. Data were shown as means ± SD (*n* = 6), *P < 0.05 compared with CTL

**Fig. 6:**
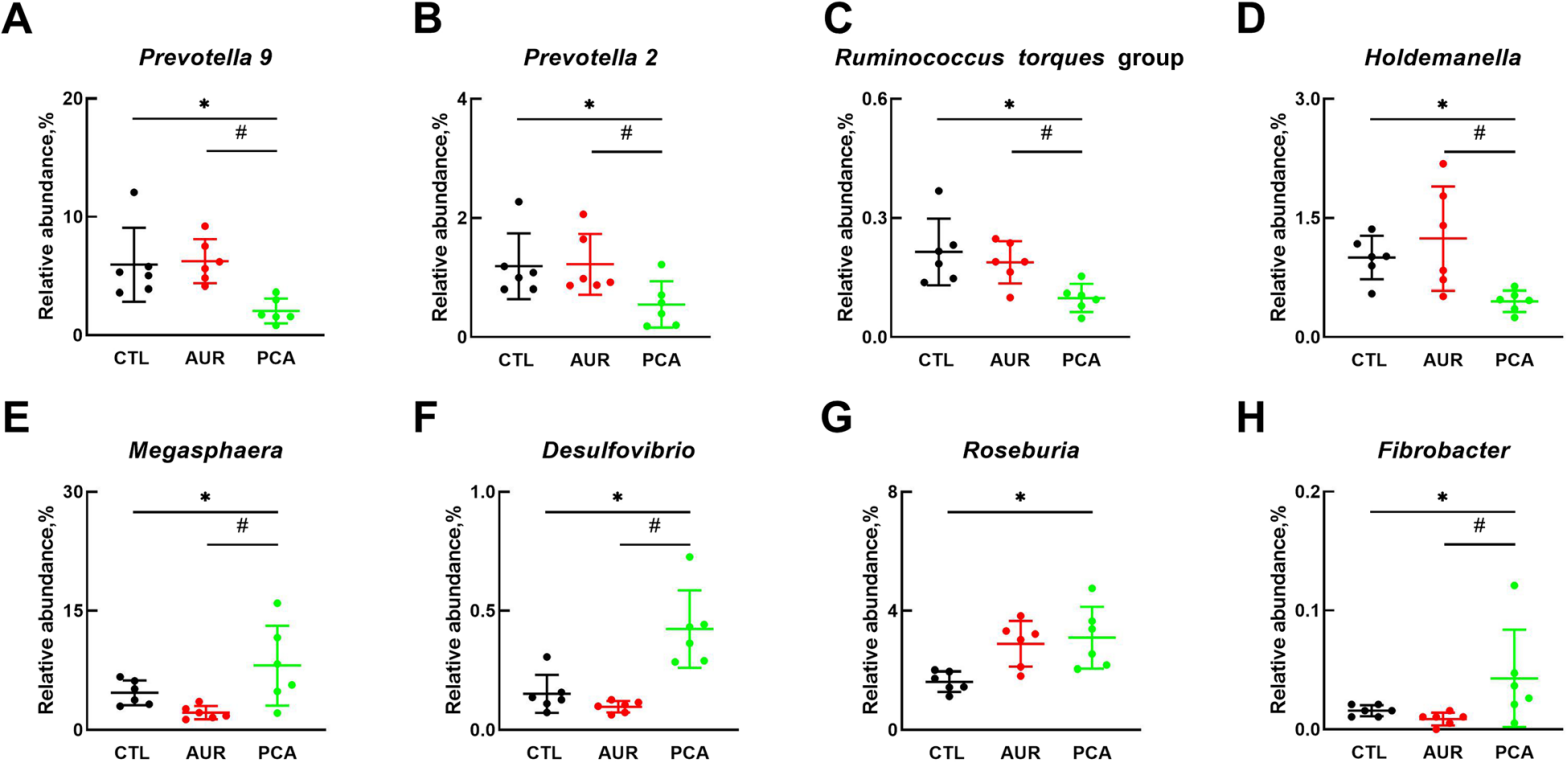
Modulation of gut microbiota by PCA at the genus level. The relative abundance of microbial genera including *Prevotella 9* (**a**), *Prevotella 2* (**b**), *Ruminococcus torques* group (**c**), *Holdemanella* (**d**), *Megasphaera* (**e**), *Desulfovibrio* (**f**), *Roseburia* (**g**), and *Fibrobacter* (**h**) in each group. CTL, a basal diet; AUR, a basal diet containing 50 mg/kg aureomycin; PCA, a basal diet supplemented with 0.4% protocatechuic acid. Data were shown as means ± SD (*n* = 6), *P < 0.05, ^#^*P* < 0.05 compared with AUR

The relative abundances of different phyla were presented in Fig. 5. The microbial community was dominated by Bacteroidetes, Firmicutes, Actinobacteria and Proteobacteria, which were more than 97% (Fig. 5A). As compared with CTL and AUR group, PCA significantly increased the ratio of Firmicutes to Bacteroidetes (Fig. 5B), by both inhibiting Bacteroidetes (Fig. 5C) and increasing the relative abundance of Firmicutes (Fig. 5D). At the genus level (Fig. 6), PCA significantly decreased the relative abundance of *Prevotella* 9 (Fig. 6A), *Prevotella* 2 (Fig. 6B), *Runminococcus torques* group (Fig. 6C) and *Holdemanella* (Fig. 6D), as compared with CTL or AUR group. On the other hand, PCA significantly increased the relative abundance of *Megasphaera* (Fig. 6E), *Desulfovibrio* (Fig. 6F) and *Fibrobacter* (Fig. 6H) as compared with CTL or AUR group, and significantly enhanced the relative abundance of *Roseburia* (Fig. 6G) as compared with CTL group.

### The correlations among antioxidant indicators, cytokines, tight junction proteins and gut microbiota

**Fig. 7:**
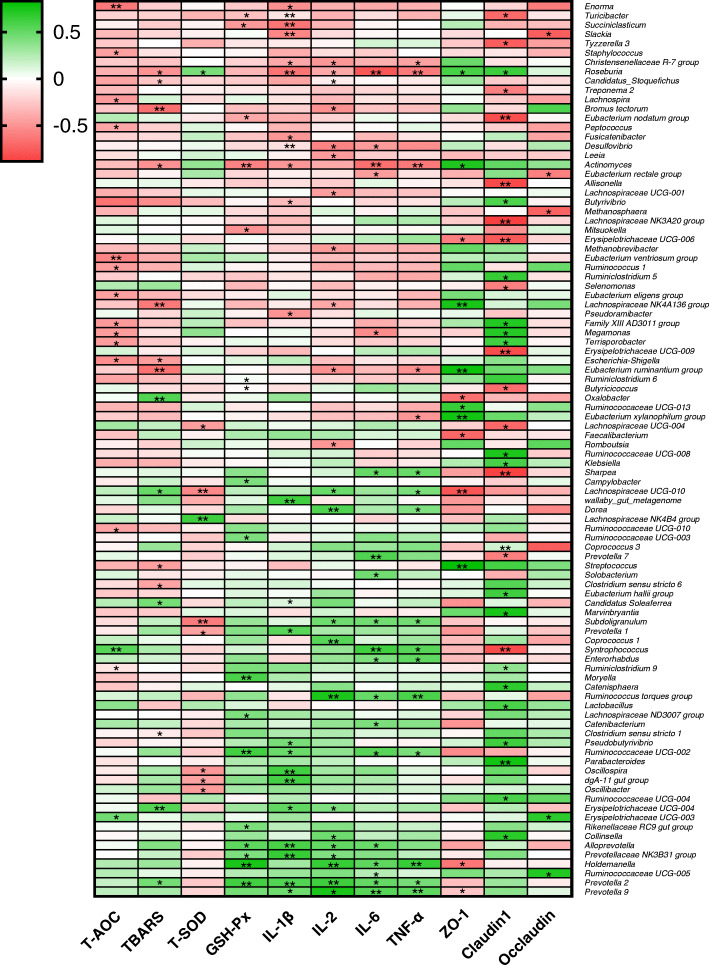
Heatmap of Spearman’s correlation between the gut microbiota and anti-oxidant indicators, inflammatory cytokines, or tight junction proteins. The intensity of the colors represented the degree of association (red, negative correlation; green, positive correlation). Significant correlations were marked by *P < 0.05, **P < 0.01

To further understand the role of whole gut microbiota in regulating antioxidant, anti-inflammatory and intestinal barrier function, their relationship was analyzed by Spearman’s correlation analysis. The results are shown in Fig. 7, a total number of 96 microbial genera were significantly correlated with the antioxidant indicators, cytokines levels or the expression of tight junction proteins. Among the microbial genera down-regulated by PCA, *Prevotella* 9 and *Prevotella* 2 had a positive correlation (*P* < 0.05) with IL-1β, IL-2, IL-6 and TNF-α levels, while *Holdemanella* and *Ruminococcus torques* group were positively correlated (*P* < 0.05) with IL-1β, IL-2 and IL-6 levels. Moreover, *Prevotella* 9 and *Holdemanella* were negatively correlated (*P* < 0.05) with the expression of ZO-1. On the other hand, the relative abundance of *Desulfovibrio*, one of the up-regulated microbial genera by PCA, had a negative correlation (*P* < 0.05) with IL-1β, IL-2 and IL-6 levels, while *Roseburia* was negatively correlated (*P* < 0.05) with IL-1β, IL-2, IL-6 and TNF-α, but positively correlated (*P* < 0.05) with the expression of ZO-1 and claudin-1. In addition, the relative abundance of *Roseburia* was positively (*P* < 0.05) correlated with SOD activity and negatively (*P* < 0.05) related to the TBARS level, which had a positive correlation (*P* < 0.05) with *Prevotella* 2.

## Discussion

Weaning of piglets can cause an oxidative balance disturbance, immune response and intestinal dysfunction [2, 3], which result in intestinal injury and damage of the intestinal epithelial barrier to cause diarrhea and systemic inflammation [4]. Excessive ROS could be produced by either bacterial endotoxin or feed ingredients because of the reduction in digestive enzymes activities during weaning. A previous study revealed that the plasma TBARS level was significantly increased 3 d after the weaning of piglets, and then returned to normal gradually [2]. In this study, LPS was administered to the piglets to induce oxidative stress and inflammation in the later stage. Our results showed that PCA and AUR significantly decreased the TBARS level indicating that they could reduce oxidative stress in piglets. Same as AUR, PCA showed no significant effect on the level of T-AOC and T-SOD, but PCA significantly decreased the GSH-Px level. Previous studies revealed that AUR may attenuate oxidative stress through the reduced expression of heat shock 27 kDa protein (HSP27), catalase and phospholipid hydroperoxide glutathione peroxidase (GPX4) in small intestine [25, 26]. Although PCA was reported to increase T-AOC level in a hypertensive rat model [27], and enhance the activity of SOD and GSH-Px in a 2,4,6-trinitrobenzenesulfonic acid (TNBS)-induced mouse colitis model [28], it potentially had a direct quenching effect on oxidative stress in this study. GSH-Px is composed mainly of glutathione (GSH), which belongs to the intracellular antioxidant system, and the function of GSH is scavenging ROS produced during normal aerobic cellular respiration [29]. However, a previous study revealed that PCA (25 mmol/L) might lower the level of intracellular GSH in human gingival epithelioid S-G cells and human squamous carcinoma HSC-2 cells [30]. To some extent, this may explain why PCA decreased the activity of GSH-Px in the present study. Another potential reason might be the modulated microbiota, since *Prevotella* 2 and *Holdemanella*, two of the main microbial genera inhibited by PCA, showed a significant positive correlation with the activity of GSH-Px (Fig. 7).

Inflammation induced by multiple factors such as oxidative stress [2], pathogenic bacteria [31] and the increased intestinal epithelial permeability [32] has been considered as the main cause of post-weaning diarrhea. During weaning, LPS from Gram-negative bacteria can activate TLR4-mediated inflammatory pathways such as nuclear factor kappa-B (NF-κB) to induce the production of inflammatory cytokines (IL-1β, IL-2, IL-6 and TNF-α) [6], which have a potential correlation with the expression of tight junction proteins [33]. In this study, both PCA and AUR significantly reduced the level of IL-6, while PCA also significantly decreased IL-2 and TNF-α level. This may partially explain the enhanced expression of ZO-1 and claudin-1, since the overproduction of IL-6 and TNF-α was considered to participate in enhancing intestinal epithelial permeability [34]. Recent studies suggested that intestinal inflammation might be associated with microbial composition changes [20]. AUR can inhibit the growth of pathogenic bacteria like *Escherichia-Shigella,* which can cause intestinal inflammation through the activation of nuclear factor-κB (NF-κB) and mitogen-activated protein kinase (MAPK) signal pathways [35, 36]. Our previous study revealed that cyanidin 3-glucoside could attenuate inflammation by regulating gut microbiota [5]. As the primary metabolites of cyanidin 3-glucoside, PCA has a potential effect on the gut microbial community [9]. In the present study, dietary supplementation of PCA increased the Firmicutes/Bacteroidetes ratio, in which Firmicutes are more effective as an energy source than Bacteroidetes in promoting more efficient absorption of calories [5, 37]. Furthermore, the decrease in the phylum Firmicutes was also observed in Crohn’s disease patients [20], and Firmicutes are considered as one of the producers of short-chain fatty acid (SCFA) [38].

At the genus level, *Prevotella* 2, *Prevotella* 9, *Holdemanella*, and *Ruminococcu torques* group were observed as the major down-regulated microbes by PCA. *Prevotella* was reported to be increased by carbohydrate-based diet and increased from nursed to weaned periods of piglets [39, 40]. The *Ruminococcus torques* group is considered as one of the bacterial species to decrease gut barrier integrity [41], and *Holdemanella* had a positive correlation with the production of IL-6, IL-1β, and TNF-α in recent studies [42]. In this study, *Prevotella* 2, *Prevotella* 9, *Holdemanella* and *Ruminococcu torques* group were all positively correlated with the production of inflammatory cytokines, while *Prevotella* 9 and *Holdemanella* were negatively correlated with the expression of ZO-1. On the other hand, *Roseburia*, *Desulfovibrio*, *Megasphaera* and *Fibrobacter* were identified as the up-regulated microbial genera by PCA. *Megasphaera* and *Roseburia* are considered as a lactate-utilizing butyrate producer that promotes the production of butyrate, which can be the energy source of intestinal epithelial cell [43, 44]. *Fibrobacter* was reported to be the dominant bacteria in fiber degradation and may also involve in the production of SCFA [38]. *Desulfovibrio* is considered as the sulfate-reducing bacteria which can promote the metabolism of sugars [45]. Our results revealed that *Roseburia* and *Desulfovibrio* were negatively correlated with the inflammatory markers, and *Roseburia* had a positive correlation with the expression of ZO-1 and claudin-1. However, *Megasphaera* and *Fibrobacter* showed no significant correlation with the antioxidant indicators, inflammatory cytokines and the expression of tight junction proteins. In addition, the microbial genera modulated by PCA were not significantly influenced by the antibiotic AUR, which suggests that PCA potentially regulated the gut microbiota in a way different from AUR. These results provided a theoretical basis for understanding the relationship between the intestinal barrier function and gut microbiota modulated by PCA, although further investigations based on metabolomics should be conducted in the future studies.

## Conclusion

In conclusion, dietary supplementation of AUR or PCA can alleviate oxidative stress (TBARS), reduce the level of IL-6, and increase the expression of tight junction proteins including ZO-1 and claudin-1 in LPS-challenged weaned piglets. Moreover, PCA can decrease the production of IL-2 and TNF-α. Analysis on gut microbiota revealed that PCA down-regulated *Prevotella* 9, *Prevotella* 2, *Holdemanella* and *Ruminococcus torques* group which promote the production of inflammatory cytokines as well as up-regulated *Roseburia* and *Desulfovibrio* which inhibit the inflammatory cytokines.

## Supplementary information

**Additional file 1: Supplemental Table 1** Composition of the basal diet.

**Additional file 2: Supplemental Table 2** GenBank accession numbers, sequences of forward and reverse primers, and fragment sizes used for real-time PCR.

## Data Availability

All data generated or analyzed during this study can be made available by the corresponding author upon reasonable request.

## Abbreviations

ADFI: Average daily feed intake
ADG: Average daily gain
ANOSIM: Analysis of similarities
AUR: Aureomycin
CAT: Catalase
CTL: Control group
F/G: Feed/gain ratio
GPX4: Phospholipid hydroperoxide glutathione peroxidase
GSH-Px: Glutathione peroxidase
GSH: Glutathione
HSP27: Heat shock 27 kDa protein
IL: Interleukin
LPS: Lipopolysaccharide
MAPK: Mitogen-activated protein kinase
NF-κB: Nuclear factor-κB
OTUs: Operational taxonomic units
PCA: Protocatechuic acid
RDP: Ribosomal database project
SCFA: Short-chain fatty acid
T-AOC: Total antioxidant capacity
TBARS: Thiobarbituric acid reactive substances
TNBS: 2,4,6-trinitrobenzenesulfonic acid
TNF-α: Tumor necrosis factor-α
T-SOD: Total superoxide dismutase
