# Supplementary material for: Dietary protocatechuic acid ameliorates inflammation and up-regulates intestinal tight junction proteins by modulating gut microbiota in LPS-challenged piglets
Source: J Anim Sci Biotechnol. 2020 Sep 9;11:92. doi: 10.1186/s40104-020-00492-9 (PMC7487840; doi:10.1186/s40104-020-00492-9)
Supplement: Supplementary file 1 — Additional file 1: Supplemental Table 1 Composition of the basal diet. [file 40104_2020_492_MOESM1_ESM.docx]

**Supplemental Table 1**, Composition of the basal diet

| Ingredients | % |
| --- | --- |
| Corn | 38.00 |
| Extruded corn | 18.50 |
| Soybean meal, 46% CP | 13.00 |
| Extruded soybean | 10.00 |
| Fish meal | 5.00 |
| Whey powder | 12.00 |
| Soy oil | 0.72 |
| CaHPO_4_ | 0.78 |
| Limestone | 1.00 |
| Salt | 0.30 |
| *L*-Lysine HCl, 98% | 0.32 |
| *DL*-Methionine | 0.16 |
| *L*-Threonine | 0.11 |
| *L*-Tryptophan | 0.01 |
| Vitamin premix^a^ | 0.03 |
| H_3_ClCu_2_O_3_ | 0.001 |
| ZnO | 0.04 |
| FeSO_4_·7H₂O | 0.0333 |
| MnSO_4_ | 0.00129 |
| KI | 0.0019 |
| Na_2_SeO_3_ | 0.006 |
| Total | 100 |
| Energy and nutrient composition |  |
| DE, kcal/kg | 3459 |
| CP, % | 19.62 |
| Ca, % | 0.93 |
| Total P, % | 066 |
| Digestible P, % | 0.42 |
| Lys, % | 1.39 |
| Met, % | 0.49 |
| Thr, % | 0.87 |
| Trp, % | 0.22 |

^a^The vitamin premix provided for per kg of feed: choline chloride, 500 mg; vitamin A, 10,500 IU; vitamin D_3_, 3,300 IU; vitamin E, 22.5 IU; vitamin K_3_, 3 mg; vitamin B_1_, 3 mg; vitamin B_2_, 7.5 mg; vitamin B_6_, 4.5 mg; vitamin B_12_, 0.03 mg; niacin, 30 mg; pantothenate, 15 mg; folic acid, 1.5 mg; biotin, 0.12 mg.
