# Supplementary material for: Dietary protocatechuic acid ameliorates inflammation and up-regulates intestinal tight junction proteins by modulating gut microbiota in LPS-challenged piglets
Source: J Anim Sci Biotechnol. 2020 Sep 9;11:92. doi: 10.1186/s40104-020-00492-9 (PMC7487840; doi:10.1186/s40104-020-00492-9)
Supplement: Supplementary file 2 — Additional file 2: Supplemental Table 2 GenBank accession numbers, sequences of forward and reverse primers, and fragment sizes used for real-time PCR. [file 40104_2020_492_MOESM2_ESM.docx]

**Supplemental Table 2** GenBank accession numbers, sequences of forward and reverse primers, and fragment sizes used for real-time PCR

| Target | GenBank number | Primer sequence | Size, bp |
| --- | --- | --- | --- |
| ZO-1 | XM 021098896.1 | F:5'-GGGGCAATCTCAACTCCTGT-3'  R: 5'-GGTTGTCCAACTTGGGCAT-3' | 137 |
| Claudin 1 | NM 001244539.1 | F:5'-CAGATATGAATTTGGTCAGGCTC-3'  R:5'-CACTGGAAGGCGAAGGTTT-3' | 149 |
| Occludin | XM 005672525.3 | F:5'-TCTCAGCCAGCGTATTCTTTC-3'  R:5'-GCACATCACGATAACGAGCAT-3' | 111 |
| Beta-actin | XM 003124280.5 | F:5'-GAGATTGGCATGGCTTTATTTG-3'  R:5'-ACTGCTGTCACCTTCACCGTT-3' | 127 |
